# Tricyclic pyrone compounds prevent aggregation and reverse cellular phenotypes caused by expression of mutant huntingtin protein in striatal neurons

**DOI:** 10.1186/1471-2202-10-73

**Published:** 2009-07-08

**Authors:** Eugenia Trushina, Sandeep Rana, Cynthia T McMurray, Duy H Hua

**Affiliations:** 1 Department of Molecular Pharmacology and Experimental Therapeutics, Mayo Clinic, 200 First St. SW, Rochester, Minnesota 55905, USA; 2 Department of Chemistry, CBC Building, Kansas State University, Manhattan, KS 66506, USA; 3 Department of Biochemistry and Molecular Biology, Mayo Clinic, 200 First St. SW, Rochester, Minnesota 55905, USA

## Abstract

**Background:**

Huntington's disease (HD) is a progressive neurodegenerative disorder caused by a CAG repeat expansion mutation in the coding region of a novel gene. The mechanism of HD is unknown. Most data suggest that polyglutamine-mediated aggregation associated with expression of mutant huntingtin protein (mhtt) contributes to the pathology. However, recent studies have identified early cellular dysfunctions that preclude aggregate formation. Suppression of aggregation is accepted as one of the markers of successful therapeutic approaches. Previously, we demonstrated that tricyclic pyrone (TP) compounds efficiently inhibited formation of amyloid-β (Aβ) aggregates in cell and mouse models representing Alzheimer's Disease (AD). In the present study, we aimed to determine whether TP compounds could prevent aggregation and restore early cellular defects in primary embryonic striatal neurons from animal model representing HD.

**Results:**

TP compounds effectively inhibit aggregation caused by mhtt in neurons and glial cells. Treatment with TP compounds also alleviated cholesterol accumulation and restored clathrin-independent endocytosis in HD neurons.

**Conclusion:**

We have found that TP compounds not only blocked mhtt-induced aggregation, but also alleviated early cellular dysfunctions that preclude aggregate formation. Our data suggest TP molecules may be used as lead compounds for prevention or treatment of multiple neurodegenerative diseases including HD and AD.

## Background

Huntington's Disease (HD) is one of several hereditary progressive neurodegenerative disorders caused by expansion of a CAG repeat in the respective disease genes. In HD, unstable CAG expansion within the coding region of the IT15 gene is translated into an abnormally long polyglutamine tract near the N-terminus of the protein called huntingtin (htt) [1-3]. While mutation analysis and transgenic animal models for disease have unequivocally identified the expanded polyglutamine tract as key in toxicity [4,5], the mechanism by which mutant htt (mhtt) progressively kills brain cells is poorly understood.

Long polyglutamine tracts are known to form hydrogen bonded, β-sheets ("polar zippers") that are prone to aggregation [6,7]. Indeed, aggregates called inclusion bodies have been identified in human disease tissue for all polyglutamine disorders [8,9]. Aggregates were found to affect vital cellular functions and accelerate cell death [10]. Suppression of aggregate formation has been shown to be beneficial in cell models for HD and is accepted as one of the markers of successful therapeutic approaches [11]. Consequently, aggregate formation has served as a phenotype in screening of small molecules and peptides for their inhibitory properties that promote cell survival in mhtt-expressing cells [12]. Screening of the NINDS Custom Collection of FDA approved drugs for their ability to prevent aggregation has produced a number of compounds including gossypol, gambogic acid, juglone, celastrol, sanguinarine and anthralin. Although each compound effectively reversed aggregation of amino terminal fragment of mutant huntingtin (1–171) with 58 polyglutamines *in vitro*, none of these molecules was effective in promoting survival of R6/2 mice, a model representing the most severe HD phenotype [12]. Thus, blocking aggregate formation, by itself, may not be a definitive predictor of the efficiency of potential therapeutics. Additionally, mhtt has multiple cellular interacting partners and confers many adverse effects in cells [13]. Since no single physiological process has yet been identified as the primary therapeutic target, better functional screening phenotypes would be necessary in order to evaluate the effectiveness of particular compounds.

**Figure 1:**
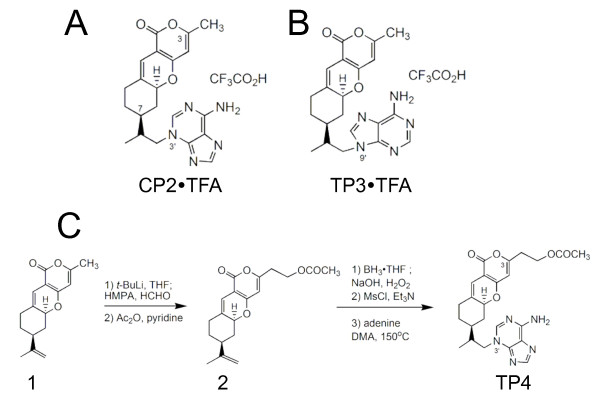
**Chemical structures of TP compounds utilized in the study and synthesis of compound TP4**. TFA – trifluoracetic acid; THF – tetrahydrofuran; HMPA – hexamethylphophoramide.

We have previously synthesized and examined the biological activity of a number of tricyclic pyrone (TP) analogs and found that TP compounds directly bind to and inhibit formation of amyloid-β (Aβ) aggregates in cell model representing Alzheimer's Disease (AD) [14-16]. Moreover, 2-week treatment with CP2 (Figure 1A) dramatically reduced formation of non-fibrillar and fibrillar Aβ oligomers *in vivo *in mouse model representing familial AD [16]. Thus, TPs represent a promising class of compounds with anti-aggregate properties. However, whether TP compounds could prevent aggregate formation in other neurodegenerative disorders has not been studied. It is also unknown whether TP compounds could ameliorate earlier cellular defects that preclude aggregate formation in the cells.

In our early study, we have established a cellular model that allowed assessment of the effect of mhtt-induced aggregate formation on survival of primary neurons, cells that are most vulnerable in HD [17]. In this model, transfection of primary neurons from control mice with GFP-tagged truncated mhtt fragment (with 82 polyglutamines) caused rapid formation of aggregates that marked late events in HD progression. Recently, we identified cellular dysfunctions that arise early in disease progression prior to aggregate formation. We demonstrated that expression of full-length mhtt causes cholesterol accumulation and inhibition of clathrin-independent caveolin-1 (cav-1)-related endocytosis in embryonic striatal neurons from HD72 mice [18,19]. These observations lead us to test whether TP compounds could effectively prevent mhtt-induced aggregation and restore early cellular defects associated with HD. We also reported synthesis and evaluation of biological activity of a new TP compound, TP4 (Figure 1C), which was modified to possess higher functionality and ability to penetrate blood-brain barrier. We found that treatment with TP compounds not only prevented aggregate formation caused by expression of truncated form of mhtt in both primary neurons and glial cells, but also reduced cholesterol accumulation and restored endocytosis inhibited by expression of full-length mhtt.

## Results

### Synthesis of a new tricyclic pyrone analogue, TP4

Previously, we found that tricyclic pyrone molecules, CP2 and TP3, inhibit formation of toxic Aβ oligomers and prevent cell death in MC65 cells conditionally expressing a partial β APP fusion protein (amino-17 residues+carboxyl-99 residues; SβC), C99 [14-16,20-22]. Structurally, CP2 and TP3 compounds consist of a tricyclic pyranopyrone skeleton and an adenine moiety attached on its N3' or N9' to the C7 isopropyl group of the fused cyclohexane ring (Figure 1A, B). The N3'-molecule, CP2, is ten times more efficient in preventing cell death in MC65 cells than the N9'-derivative TP3 [14]. However, the bioactivity of TP compounds where the methyl group at C3 atom will be substituted with other groups that could enhance their solubility in water has not been examined. To investigate the effect of C3-substituent, we synthesized a CP2 analogue, TP4 (Figure 1C), possessing an acetoxyethyl group at C3 and compared its biological activity with that of CP2 and TP3. We previously synthesized CP2 and TP3 compounds via a sequence of reactions starting from compound **1 **(Figure 1C), derived from a one-pot condensation reaction of 4-hydroxy-6-methyl-2-pyrone and (*S*)-(-)-perillaldehyde [22]. Compound TP4 was similarly synthesized from compound **1 **(Figure 1C) by a deprotonation with lithium diisopropylamide (LDA) followed by formaldehyde and then acetic anhydride (Ac_2_O) to give compound **2**. It should be noted that the hydroxyl intermediate derived from the reaction of compound **1 **with formaldehyde is an unstable compound, which undergoes reverse aldol reaction to produce compound **1 **and formaldehyde under weakly acidic conditions. Hence, protection of the hydroxyl function with an acetyl moiety is needed. Selective hydroxylation of compound **2 **with boraneTHF followed by oxidation with NaOH-H_2_O_2_, mesylation of the resulting hydroxyl function with methanesulfonyl chloride (MsCl), and displacement with adenine in *N, N*-dimethylacetamide (DMA) afforded TP4. CP2, TP3 and TP4 were purified using HPLC with acetonitrile, water and trifluoroacetic acid (TFA) (1%) as solvents. The pure solids obtained after lyophilization were stable water-soluble TFA salts.

### Treatment with TP compounds at low concentrations does not cause toxicity in embryonic neurons

**Figure 2:**
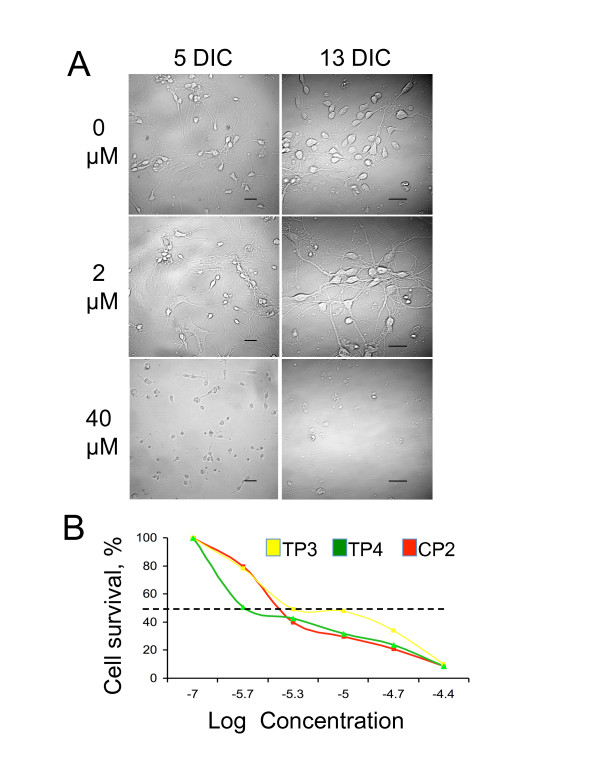
**Treatment with TP compounds at low concentrations is not toxic for primary neuronal cultures**. A. Survival of primary striatal neurons from control mice treated with different concentrations of CP2. Days in culture (DIC) and CP2 concentrations are indicated. Images were acquired using LSM 510 confocal microscope with 63 × or 100 × oil DIC lenses (1.4 na). Scale bars, 20 um. **B**. Lethal dose at which 50% of the neurons died (LD_50_) for all three TP compounds was estimated at day 6 after plating using LIVE/DEAD assay.

Effective pharmacological compounds must be non-toxic to cells. To evaluate the toxicity of TP compounds, primary embryonic (E17) striatal neurons from FVB control mice were cultured and treated next day after plating with 0, 2, 5, 10, 20 and 40 μM of different TPs. Cells were kept under these conditions for 13 days. Every other day, the neuronal morphology and the extent of cell death were evaluated by imaging of five randomly selected fields (Figure 2A). For all TP compounds tested, concentrations below 5 μM did not cause significant cell death for up to 13 days in culture (Figure 2A, 2 μM). About 60% of neurons remain healthy and preserve their morphology. However, concentrations above 5 μM effectively killed cells in less than 7 days (Figure 2A, 40 μM). At day 6 in culture, for each TP compound, we determined the lethal dose at which 50% of the cells died (LD_50_) (Figure 2B). The results suggested concentrations between 2 and 5 μM were least toxic for all TPs tested. Survival of HD neurons in response to TP treatments did not differ from control cells (data not shown). Thus, we used these conditions in the following experiments.

### Treatment with CP2 inhibits aggregation in primary embryonic neurons expressing mhtt

**Figure 3:**
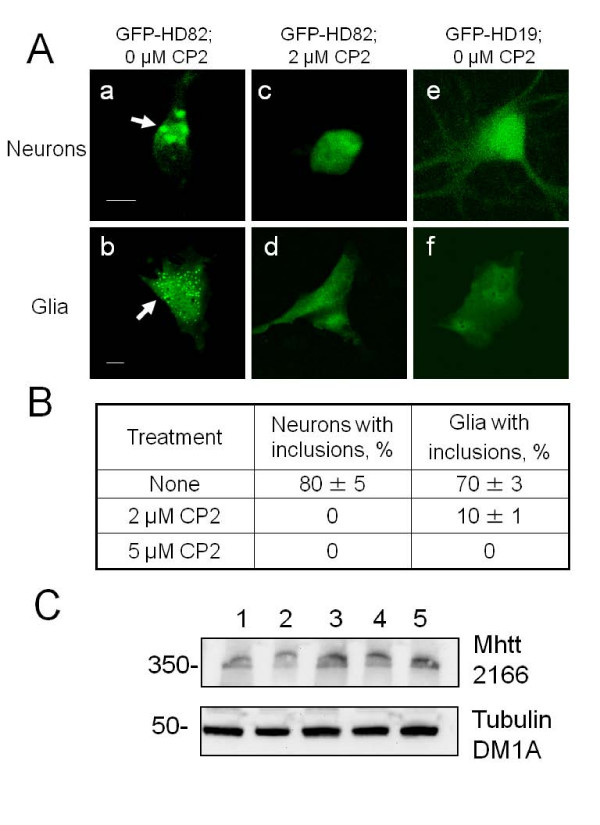
**Treatment with CP2 prevents aggregate formation caused by expression of truncated form of mhtt in neurons and glial cells**. **A**. Transfection with truncated N-terminal form of mhtt with 82 polyglutamines (GFP-HD82) causes formation of aggregates (arrows) in striatal neurons (**a**) and glial cells (**b**) from control mice. Expression of htt fragment with 19 polyglutamines (GFP-HD19) does not cause formation of aggregates (**e, f**). Pre-treatment with 2 μM of CP2 prevents formation of aggregates in cells transfected with GFP-HD82 (**c, d**). Images of live cells were taken using LSM 510 confocal microscope with 100× oil DIC lens (1.4 na). Scale bar, 10 μm. **B**. Quantification of aggregate formation in neurons and glial cells from control mice with and without CP2 treatment. **C**. Treatments with TP compounds do not affect the expression of mhtt in primary neurons from HD mice. Neurons (E17) from HD mouse were treated with different doses of CP2, TP3, and TP4 for 6 days. Western Blot analyses were performed using specific monoclonal anti-huntingtin antibody 2166 (1:3000, Chemicon). Lane 1 – HD neurons, no treatment; lane 2 – HD neurons treated with 2 μM CP2; lane 3 – HD neurons treated with 5 μM CP2; lane 4 – HD neurons treated with 2 μM TP3; lane 5 – HD neurons treated with 2 μM TP4. Anti-tubulin antibody (DM1A, 1:6000, Sigma) was used as loading control.

Previously, we have shown that treatment with CP2 and TP3 effectively prevent Aβ aggregation in cell and animal model for AD [14,16]. Therefore, we tested whether TP compounds can also prevent aggregation caused by expression of mhtt. It has been well documented, that expression of a truncated form of mhtt with expanded polyglutamine region leads to rapid formation of intracellular aggregates [17]. Indeed, transfection of neuronal cultures from control mice with GFP-HD82, a truncated form of human htt (amino acid 1–221) with pathologic 82 polyglutamine repeats, leads to rapid formation of visible aggregates in both primary striatal neurons and glial cells within 2–10 hours post transfection (Figure 3A, a, &3A, b ). Aggregates are visible as green puncta indicated with arrows). However, treatment of striatal cultures with CP2 from the day of plating eliminated formation of inclusions in neurons and reduced aggregate formation in glial cells by 90% relative to untreated cells (Figures 3A, c &3A, d, &3B). Aggregation was specific to expression of GFP-mhtt with expanded polyglutamine tract since no aggregates were formed in cells transfected with control plasmid expressing a short GFP-htt fragment with 19 polyglutamines (non-pathologic length, GFP-HD19) (Figure 3A, e &3A, f). Thus, CP2 efficiently inhibited aggregation caused by expression of truncated form of mhtt in neurons and glial cells at the concentrations as low as 2 μM.

### Treatments with TP compounds do not affect the expression of mhtt

Control experiments were performed to examine the effects of TP compounds on the expression of mhtt. We plated neurons from HD mouse and treated cells with different doses of CP2 (2 and 5 μM), TP3 (2 μM), and TP4 (2 μM) for 6 days starting from the first day of plating. Treated and untreated HD cells were collected, lysed, and subjected to the Western Blot analysis using specific monoclonal anti-huntingtin antibody 2166 (1:3000, Chemicon) as previously described [17,18]. Results are shown in Figure 3C and data indicated that treatments with TP compounds do not affect expression of mhtt in primary neurons from HD mice (similarly, TP compounds do not affect the expression of cav-1; data not shown).

### Treatment with CP2 restores clathrin-independent endocytosis inhibited by expression of mhtt

Since aggregate formation is a late event in disease progression, we next investigated whether CP2 treatment could alleviate early cellular dysfunctions associated with HD. Recently, we have shown that expression of full-length mhtt causes abnormal cholesterol accumulation and inhibition of clathrin-independent caveolin-related endocytosis in embryonic striatal neurons from HD72 mice early in disease progression prior to aggregate formation [18]. Thus, we first tested whether CP2 treatment could restore defective endocytosis associated with mhtt expression in striatal neurons from HD72 mice.

**Figure 4:**
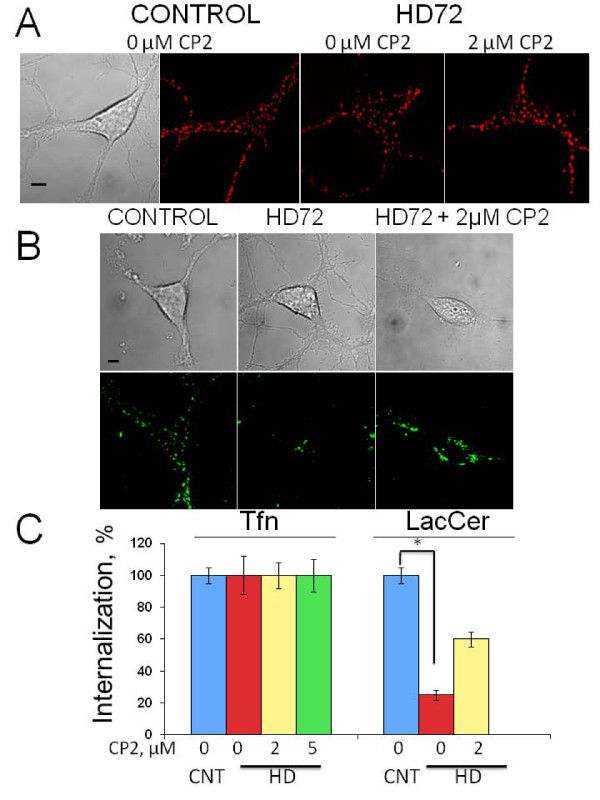
**Treatment with CP2 restores caveolin-related endocytosis inhibited by mhtt expression**. **A**. Treatment with CP2 does not affect internalization of fluorescently labelled Tfn (red) in control or HD neurons. Images represent internalization of Tfn in live neurons 10 min after addition to the cells. Tfn internalization and localization were indistinguishable in untreated and CP2-pretreated control and HD cells. Data for 2 μM CP2 treatment is shown. Scale bar, 5 μm. **B**. Internalization of LacCer (green) is inhibited in striatal HD72 neurons (HD72, middle panel) comparing to control neurons. Treatment with 2 μM CP2 restores internalization of LacCer in HD72 neurons (HD72+2 μM CP2). Images of live cells in **A **and **B **were acquired using LSM 510 confocal microscope with 100× oil DIC lens (1.4 na). Scale bar, 5 μm. **C**. Quantification of Tfn and LacCer internalization in control and HD72 striatal neurons with and without CP2 treatment acquired in experiments shown in **A **and **B**. At least 10 cells were taken into analysis in each 3 independent experiments. *, p < 0.001.

Internalization of essential extracellular components in neurons occurs through multiple endocytic pathways including clathrin-dependent and clathrin-independent caveolin-related endocytosis [23]. In order to evaluate whether CP2 treatment could restore defective endocytosis in HD72 neurons, we monitored internalization of fluorescently labelled cargoes specific for each pathway. Alexa Fluor 594-labeled transferrin, Tfn, was used as a marker for clathrin-mediated endocytosis [24], and BODIPY-lactosylceramide, LacCer, as a marker for clathrin-independent caveolin-related endocytosis [25]. Consistent with previous observations, expression of mhtt had no effect on Tfn internalization in HD72 neurons comparing to control cells (Figure 4A, C). Treatment with either 2 or 5 μM of CP2 also did not affect Tfn internalization or intracellular localization in HD72 neurons comparing to control or untreated HD72 cells (Figure 4A, C). These data suggest that neither CP2 treatment nor mhtt expression affected clathrin-mediated endocytosis in striatal neurons. In contrast, the uptake of LacCer was inhibited by 70% in HD neurons relative to control cells (Figure 4B, C). However, pre-treatment with 2 μM of CP2 restored the uptake of LacCer in HD cells to approximately 60% of that in control neurons (Figure 4B, C). Thus, CP2 partially restores lipid trafficking defect caused by expression of mhtt, and the beneficial effect of CP2 is evident prior to visible formation of aggregates.

### Treatment with TP compounds prevents cholesterol accumulation in neurons caused by expression of mhtt

**Figure 5:**
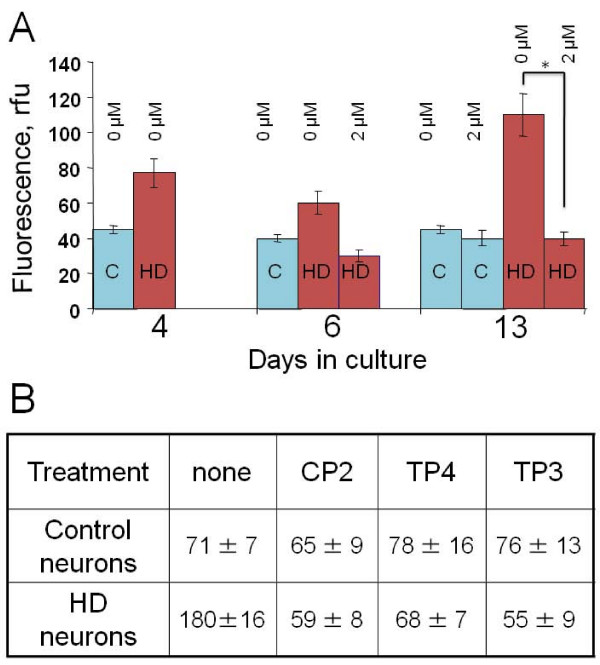
**Treatment with CP2 and other TP compounds prevents cholesterol accumulation in HD neurons**. **A**. Intracellular cholesterol levels in striatal neurons from control (C) and HD72 (HD) mice with and without CP2 treatment at different days in culture. Control neurons do not accumulate cholesterol; HD neurons without CP2 treatment accumulate cholesterol to high extent than control neurons (13 DIC). Treatment with CP2 prevented cholesterol accumulation in HD neuron at 13 DIC. At least 15 cells were taken into analysis for each data point. *, p < 0.001. **B**. Different TP compounds prevent cholesterol accumulation in HD neurons with similar efficiency. Data represent intracellular cholesterol levels in untreated control and HD neurons and neurons treated with 2 μM of different TP compounds on day 13 in culture. Numbers represent relative fluorescence units of filipin staining. At least 10 cells in each experiment were taken into analysis.

Since CP2 has been found to restore lipid trafficking in primary striatal neurons expressing mhtt, we next investigated whether CP2 and its analogues could also avert another early cellular defect found in HD neurons, the accumulation of cholesterol [18]. To test this, we plated embryonic (E17) striatal neurons from control and HD72 mice and immediately treated them with 2 μM of different TP compounds. Neurons were kept under continues TP treatment for 13 days, then cells were fixed and intracellular levels of free cholesterol were estimated and compared to untreated neurons using filipin staining. Filipin is an antibiotic that specifically binds free cholesterol and could be visualized under the UV light [26]. Consistent with our previous data, untreated HD72 neurons accumulated significant amounts of cholesterol after 13 days in culture relative to control neurons (Figure 5A, B). In contrast, in HD72 neurons kept in the presence of 2 μM CP2 cholesterol levels did not increase and were similar to control neurons 13 days after plating (Figure 5A, Day 13). Thus, CP2 treatment effectively prevented accumulation of cholesterol caused by mhtt expression.

Next, we compared the efficiency of different TP compounds in preventing cholesterol accumulation in embryonic striatal HD neurons (Figure 5B). In control neurons, pre-treatment with CP2, TP3 or TP4 did not affect cholesterol levels as measured using filipin 13 days after plating (Figure 5B, control neurons). However, treatment with 2 μM of TP3 or TP4 was as efficient in lowering cholesterol in HD neurons as treatment with CP2 (Figure 5B, HD neurons). All TP compounds effectively prevented cholesterol accumulation in HD neurons reducing it to the levels observed in control cells. Thus, TP compounds appeared not only to efficiently prevent aggregation caused by expression of mhtt, but also suppress early cellular dysfunctions in HD neurons that precede aggregate formation.

## Discussion

The use of small molecules to inhibit specific protein-protein interactions and prevent aggregation has important potential therapeutic application [27,28]. The fact that in most neurodegenerative disorders faulty proteins form fibrils with very similar structure suggests that it is feasible to find the molecular approach that could be effectively applied in many disease [29]. However, formation of detectable aggregates usually occurs later in disease progression and may not represent the primary cause of cellular dysfunction. Therefore, it is important to evaluate whether specific treatment modalities could not only inhibit aggregation, but also alleviate cellular dysfunctions that occur early in disease progression.

Previously, we reported that tricyclic pyrone molecules, CP2 in particular, directly bind Aβ 42 oligomers and prevent formation of Aβ fibrils in cellular and mouse models for AD [15,16]. Here, we demonstrate that TP compounds also efficiently block aggregate formation in HD. Pre-treatment with CP2, the most efficient aggregate inhibitor in the AD cell model, blocked formation of aggregates caused by expression of truncated form of mhtt in both cultured primary striatal neurons and glial cells. Moreover, treatment with TP compounds alleviated early cellular defects associated with HD that preclude aggregate formation. Specifically, TP treatment partially restored clathrin-independent endocytosis and eliminated cholesterol accumulation in mhtt-expressing neurons. It will be important to test whether TP compounds demonstrate similar efficacy *in vivo *in animal models representing HD.

Htt is ubiquitously expressed with highest levels in neurons [30-32]. Both htt and mhtt interact with a large variety of cellular proteins involved in cytoskeletal dynamics [33,34], clathrin-dependent and independent endocytosis [18,35], axonal transport [36-38], postsynaptic signaling [13] and transcription [39]. Expansion of the polyglutamine repeat in htt alters its interactions with cellular proteins disrupting their functions and contributing to pathology [40,41]. In our previous study, we demonstrated that application of cav1 siRNA restored clathrin-independent endocytosis and eliminated cholesterol accumulation in HD neurons suggesting that interaction between mhtt and cav-1 underlies these cellular defects [18]. In the present experiments, treatment with TP compounds did not affect cellular levels of mhtt or cav-1 in neurons (data not shown). Thus, it is feasible that TP compounds could bind mhtt and not only prevent its aggregation but also block mhtt interaction with its targets. Preventing aberrant interactions could be beneficial, however additional experiments to demonstrate the specificity and efficiency of this approach are necessary.

Originally, TP compounds were synthesized based on the structures of pyripyropene A, an inhibitor of acyl-CoA:cholesterol acyltransferase (ACAT) [42]. ACAT is an endoplasmic reticulum-resident enzyme that regulates intracellular cholesterol homeostasis by converting excess free cholesterol to cholesteryl esters [43]. ACAT inhibitors have been shown to effectively suppress generation of Aβ, the major toxic component of senile plaques in AD, the disease where cholesterol is known to be an important risk factor [44-47]. One of the explanations of the reduced free cholesterol levels in the cells is the demonstration that ACAT inhibitors increase the expression of cholesterol efflux transporter ATP-binding cassette transporter 1 (ABCA1) [48,49]. However, the molecular mechanism of ACAT action remains to be elucidated. We found that all three TP compounds used in this study, while only partially restoring clathrin-independent endocytosis, effectively reversed mhtt-induced cholesterol accumulation in HD neurons. These observations suggest that TP compounds possess a complex mechanism of action. First, they may act as small molecules that directly bind faulty proteins preventing aggregation and blocking protein-protein interactions. Additionally, they may also affect cholesterol homeostasis by regulating cellular cholesterol efflux.

## Conclusion

In summary, we have demonstrated that TP compounds effectively inhibit mhtt-induced aggregation in HD neurons and glial cells. Additionally, treatment with TP compounds alleviated cholesterol accumulation and restored clathrin-independent endocytosis, early neuronal defects caused by expression of full-length mhtt that preclude aggregate formation. Our data suggest TP compounds may be used as lead molecules for prevention or treatment of neurodegenerative diseases including HD and AD.

## Methods

### Animals and neuronal cell cultures

All procedures involving animals were approved by the Institutional Animal Care and Use Committee. The following mouse models were used: control FVB/N [50] with 7 glutamines in mouse endogenous htt homologue and HD transgenic YAC (yeast artificial chromosome) model expressing full-length human HD cDNA containing 72 polyglutamines (HD72) [19]. Preparation and culturing of primary striatal neurons were performed as described in Supplemental Material in [17]. Briefly, mice were anesthetized with ether on gestational day 17 and fetuses were rapidly removed. Fetal brains were extracted and placed in sterile HEPES- buffered saline (HBS) (pH 7.3). The ventral part of the medial ganglionic eminence (the developmental precursor to the striatum) was dissected under a microscope in sterile conditions. Tissue was placed in 2 mg/1 ml papain (Warthington, NJ) in HBS for 20 min at 37°C. After two washes in HBS, the dissociated tissue was triturated in Dulbecco's modified Eagle's medium containing 10% Ham's F12 with glutamine (Gibco/BRL, Grand Island, NY), 10% heat inactivated fetal calf serum (Hyclone Laboratories Logan, UT) and 1× pen/strep antibiotic mixture. Cells were counted, diluted to 3 × 10^5 ^cells/ml, and 2 ml of this stock was placed in each well of a 6-well dish containing glass coverslips coated with poly-L-ornithine (1 mg/2 ml sterile borate buffer, pH 8.4). Plated cells were maintained in an incubator with 5% CO_2 _at 37°C. After 72 h in culture medium containing serum was replaced with a serum-free Neurobasal-based medium (without glutamine, Gibco/BRL, Grand Island, NY) containing 1 × pen/strep antibiotic mixture and 1 × B27 supplement (Gibco/BRL, Grand Island, NY). Quantification of neurons and glia using specific antibody staining (GFAP for astrocytes and neuron specific βIII-tubulin) demonstrates that neurons represent 95% of cells present on the coverslip. Such uniform neuronal cultures could be obtained by substituting serum (+) medium for Neurobasal medium that was specifically designed to support neuronal development and suppress glia proliferation (glial growth was found to be less than 0.5%) [51]. In cases where experiments required especially pure neuronal cultures, cells were treated with cytosine β-D-arabinofuranoside (Ara-C, Sigma, MO) to a final concentration of 2 μM after 3 and 5 days in culture to suppress proliferation of the glial cells. Such conditions allowed obtaining fully developed pure striatal neurons exhibiting synaptic activity as judged by staining with synapsin antibody and EM examination of synaptic contacts [18,36]. All experiments were performed in neurons 6 to 7 days in culture unless specifically stated.

### Synthesis of CP2 and TP3 compounds

Compounds CP2 and TP3 were synthesized as described earlier. Briefly, a selective hydroboration of compound **1 **with borane followed by hydrogen peroxide, mesylation with methanesulfonyl chloride, and displacement with adenine provided CP2 or TP3 [22].

### Synthesis of compound TP4

Synthesis of (5a*S*,7*S*)-3-(2-acetoxyethyl)-7-isopropenyl-1*H*,7*H*-5a,6,8,9-tetrahydro-1-oxopyrano [4,3-b][1]benzopyran (compound 2, Figure 1). To a cold (-10°C) solution of 0.39 g (3.88 mmol) of diisopropylamine in 10 ml of THF under argon was added 2.4 ml (3.88 mmol; 1.6 M solution in hexanes) of *n*-butyllithium via syringe, and the solution was stirred for 1 hour providing a LDA solution. In another flask, a solution of 1.00 g (3.88 mmol) of compound **1 **(Figure 1) [22] in 5 ml of THF under argon was cooled to -78°C. The LDA solution was added to the pyrone solution at -78°C via cannula, followed by 0.83 g (4.65 mmol) of HMPA. After 3 h of stirring, the solution was added to a cold (-78°C) mixture of 1.16 g (38 mmol) of paraformaldehyde in 5 ml of THF via cannula, and stirred for 2 h. The reaction solution was warmed to 25°C, diluted with 40 ml of aqueous ammonium chloride, and extracted three times with dichloromethane. The combined organic layer was washed with 40 ml of brine, dried (MgSO_4_), concentrated, and subjected to column chromatography on silica gel using a gradient mixture of hexane and ethyl acetate as eluant to give 0.15 g (15% recovery) of compound **1 **(Figure 1) and 0.32 g (28% yield) of (5a*S*,7*S*)-3-(2-hydroxyethyl)-7-isopropenyl-1*H*,7*H*-5a,6,8,9-tetrahydro-1-oxopyrano [4,3-b][1]benzopyran. [α]_D_^23 ^= +23.7° (c 3.5, CHCl_3_); ^1^H NMR δ (ppm) 6.06 (s, 1 H), 5.86 (s, 1 H), 5.11 (dd, *J *= 11, 4.4 Hz, 1 H, C5a-H), 4.75 (s, 1 H, = CH_2_), 4.73 (s, 1 H, = CH_2_), 3.90 (t, *J *= 6.2 Hz, 2 H, CH_2_O), 2.69 (t, *J *= 6.2 Hz, CH_2_), 2.48 (m, 1 H), 2.20 ~1.70 (a series of m, 5 H), 1.73 (s, 3 H, Me), 1.34 ~1.25 (m, 1 H); ^13^C NMR δ (ppm) 163.3, 162.1, 148.0, 132.8, 109.9, 109.1, 101.1, 98.2, 79.6, 59.6, 43.6, 40.0, 37.3, 32.6, 32.1, 30.5, 20.9. HRMS calculated for C_17_H_21_O_4 _(M+H) 289.1440, found 289.1411.

A solution of 0.20 g (0.70 mmol) of the above compound and 0.14 g (1.40 mmol) of acetic anhydride in 3 ml of pyridine was stirred at 25°C under argon for 7 h. The solution was diluted with ethyl acetate and washed with 1 N HCl, aqueous NaHCO_3_, and brine, dried (MgSO_4_), concentrated, and subjected to column chromatography on silica gel using a gradient mixture of hexane and ether as eluants to give 0.14 mg (62% yield) of compound **2**. [α]_D_^23 ^= +16.9° (c 0.15, CHCl_3_); ^1^H NMR δ (ppm) 6.10 (s, 1 H), 5.79 (s, 1 H), 5.13 (dd, *J *= 11.2, 4.8 Hz, 1 H, C5a-H), 4.75 (s, 1 H, = CH_2_), 4.73 (s, 1 H, = CH_2_), 4.33 (t, *J *= 6.4 Hz, 2 H, CH_2_O), 2.76 (t, *J *= 6.4 Hz, CH_2_), 2.48 (m, 1 H), 2.20 ~1.70 (a series of m, 5 H), 1.74 (s, 3 H, Me), 1.28 (qd, *J *= 12.8, 4 Hz, 1 H); ^13^C NMR δ (ppm) 170.9, 163.0, 160.9, 147.9, 132.9, 110.0, 109.6, 100.8, 98.5, 79.6, 60.8, 43.6, 40.0, 33.6, 32.5, 32.1, 30.5, 20.1, 20.9. HRMS calculated for C_19_H_23_O_5 _(M+H) 331.1545, found 331.1536.

(5a*S*,7*S*)-3-(2-Acetoxyethyl)-7-(2-hydroxy-1-methylethyl)-1*H*,7*H*-5a,6,8,9-tetrahydro-1-oxopyrano [4,3-b][1]benzopyran. To a cold (-78°C) solution of 0.14 mg (0.43 mmol) of compound **2 **in 3 ml of THF under argon, was added 0.43 ml (0.43 mmol) of BH_3_THF (1.0 M in THF), and the solution was stirred at -78°C for 30 min and kept at -20°C for 2 days. To the solution (0°C), 1 ml of water, 0.5 ml of 0.1% NaOH aqueous solution, and 0.5 ml of 30% H_2_O_2 _were added, and the resulting solution was stirred for 1 h. The solution was extracted with dichloromethane three times, the combined extract was washed with aqueous NH_4_Cl, water, and brine, dried (MgSO_4_), concentrated, and column chromatographed on silica gel using a gradient mixture of hexane and diethyl ether as eluants to give 72 mg (56% yield; based on unreacted compound **2**) of the title compound as a mixture of two diastereomers (stereochemistry differs at the newly created side chain carbon; not separable by silica gel column chromatography) along with 20 mg (14% recovery) of compound **2**. ^1^H NMR δ (ppm) 6.07 (s, 1 H), 5.78 (s, 1 H), 5.10 (m, 1 H, C5a-H), 4.32 (t, *J *= 6.2 Hz, 2 H, CH_2_O), 3.56 (m, 2 H, CH_2_OH) 2.76 (t, *J *= 6.2 Hz, CH_2_), 2.47 (m, 1 H), 2.17 ~1.10 (a series of m, 7 H), 0.91 (d, *J *= 7 Hz, 1 H); ^13^C NMR δ (ppm) 170.9, 163.0, 160.9, 133.4, 118.1, 109.3, 100.8, 98.5, 79.9, 66.0, 60.8, 40.2, 39.5, 37.6, 37.5, 37.3, 33.6, 31.2, 21.0. HRMS calculated for C_19_H_25_O_6 _(M+H) 349.1651, found 349.1649.

(5a*S*,7*S*)-3-(2-Acetoxyethyl)-7- [(2-methanesulfonyloxy)-1-methylethyl]-1*H*,7*H*-5a,6,8,9-tetrahydro-1-oxopyrano [4,3-b][1]benzopyran. A solution of 85 mg (0.24 mmol) of the above alcohol, 74 mg (0.73 mmol) of triethylamine and 42 mg (0.37 mmol) of methanesulfonyl chloride (MsCl) in 3 ml of dichloromethane was stirred under argon at 25°C for 3 h, diluted with aqueous sodium bicarbonate, and extracted twice with dichloromethane. The combined extract was washed with brine, dried (anhydrous Na_2_SO_4_), concentrated, and column chromatographed on silica gel using a gradient mixture of hexane and diethyl ether as eluants to give 94 mg (90% yield) of the title compound. ^1^H NMR δ (ppm) 6.05 (s, 1 H), 5.76 (s, 1 H), 5.07 (m, 1 H, C5a-H), 4.29 (t, *J *= 6.3 Hz, 2 H, CH_2_O), 4.10 (m, 2 H, CH_2_OS), 3.00 (s, 3 H, CH_3_S), 2.73 (t, *J *= 6.3 Hz, CH_2_), 2.47 (d, *J *= 14 Hz, 1 H), 2.17 ~1.10 (a series of m, 7 H), 2.02 (s, 3 H, CH_3_CO), 0.96 (d, *J *= 7 Hz, 1 H); ^13^C NMR δ (ppm) 170.9, 162.9, 162.3, 161.1, 132.7, 109.7, 100.7, 98.4, 79.4, 72.3, 60.8, 39.1, 37.6, 37.5, 37.4, 33.6, 32.3, 30.9, 13.3. HRMS calculated for C_20_H_27_O_8_S (M+H) 427.1426, found 427.1434.

(5a*S*,7*S*)-3-(2-Acetoxyethyl)-7- [(1*R*) and (1*S*)- 2-(*N*3-adenyl)-1-methylethyl]-1*H*,7*H*-5a,6,8,9-tetrahydro-1-oxopyrano [4,3-b][1]benzopyran (TP4). A solution of 94 mg (0.22 mmol) of (5a*S*,7*S*)-3-(2-acetoxyethyl)-7- [(2-methanesulfonyloxy)-1-methylethyl]-1*H*,7*H*-5a,6,8,9-tetrahydro-1-oxopyrano [4,3-b][1]benzopyran and 32 mg (0.24 mmol) of adenine in 1.5 ml of *N, N*-dimethylacetamide (DMA) was stirred under argon at 150°C for 7 h, and cooled to 25°C. DMA was removed under reduced pressure (70°C/0.5 mm Hg), and the residue of the distillation was triturated with 5 ml of dichloromethane. To the residue, 50 mg (0.59 mmol) of NaHCO_3 _and 3 ml of ethanol were added, and the mixture was subjected to a silica gel column chromatography using a gradient mixture of dichloromethane and ethanol as eluant to give TP4. ^1^H NMR δ (ppm) 8.07 (s, C8'H of adenine), 8.01 & 8.00 (2s, 1 H, C2'H of adenine; 2 diastereomers), 6.10 (s, 1 H, C10H), 5.79 & 5.78 (2s, 1 H, C4H), 5.05 (m, 1 H, C5aH), 4.54 (2dd, *J *= 13.5, 6.5 Hz, 1 H, CHN; 2 diastereomers), 4.34 & 4.33 (2t, *J *= 6.2 Hz, 2 H, CH_2_O; 2 diastereomers), 4.07 (dd, *J *= 13.5, 8 Hz, 1 H, CHN), 2.78 & 2.76 (2t, *J *= 6.2 Hz, 2 H, CH_2_; 2 diastereomers), 2.07 & 2.06 (2s, 3 H, Me; 2 diastereomers), 2.60 ~1.22 (a series of m, 8 H), 0.91 (d, *J *= 7.0 Hz, 3 H, Me). ^13^C NMR δ (ppm) 182.0, 181.6, 164.8, 163.3, 161.1, 155.8, 152.9, 148.0, 141.6, 131.1, 129.6, 129.1, 121.3, 100.7, 79.1, 78.9, 63.8, 59.2, 50.5, 33.6, 29.9, 21.1, 15.1. The product was further purified on a HPLC using acetonitrile, water, and TFA (1%) as solvents to give pure TP4TFA salt. HRMS calculated for C_24_H_28_N_5_O_5 _(M+H) 466.2090, found 466.2081.

### Plasmid Construction and Transfection

Experiments were performed as described in [17]. Control and mhtt plasmids were derived from insertion of sequence into pEGFP-C1 vector (Clontech). GFP-HD constructs were generated by inserting truncated (amino acids 1–221) HD cDNA that contains 19 (HD19) or 82 (HD82) CAG repeats, respectively (generous gift from C. A. Ross). All plasmids were confirmed by sequencing. Primary striatal neurons from control mice were transfected using Lipofectamine™ 2000 reagent (Invitrogen, Carlsbad, CA) and Nupherin™-neuron (BIOMOL, Plymouth Metting, PA) according to the manufacturer's instructions. Briefly, embryonic neurons (E17) were plated on glass coverslips and cultured for 6 days. Half of the cells were kept under CP2 from the first day of plating. Expression plasmids (4 μg) were mixed with 20 μl of Nupherin-neuron in 380 μl of serum-free phenol red-free DMEM. After 30 min of incubation, DNA mixture was added to lipofectamine solution in DMEM at a ratio of 4:1 (liposome to DNA), incubated for another 45 min and added to the cells for 4 hrs. Cells were washed 3 times with DMEM and maintained in the incubator at 37°C and 5% CO_2_. CP2-containing media was used in transfection experiments with cells that had been kept under CP2 prior to transfection. Cells were observed for formation of inclusions using LSM 510 laser scanning microscope (C. Zeiss, Germany) with 100× oil DIC lens (1.4 na). Imaging of the same cells over time (2 days, every 6 hours) was performed using microscopic incubator chamber with 5% CO_2 _and 37°C. Software allowed recording the position of the cells to ensure consecutive imaging of the same cell over time. At least 10 cells were taken into analysis from at least three independent experiments.

### Western blot analysis

Neurons from HD mice were plated and immediately treated with different doses of CP2 (2 and 5 uM), TP3 (2 uM) and TP4 (2 uM). Cells were kept under these conditions for 6 days. Treated and untreated HD cells were collected, lysed and subjected to the Western Blot analysis on 4–15% gradient SDS-Tris gels with 30 μg of protein loaded in each well. Htt and mhtt were detected using specific monoclonal anti-huntingtin antibody 2166 (1:3000, Chemicon). Monoclonal Tubulin DM1A antibody (1:6000, Sigma) was used for loading control. Horseradish peroxidase-conjugated anti-mouse (1:24000) secondary antibody was used.

### Filipin staining in neurons

Striatal neurons from control and HD72 mice were plated on poly-L-ornithine covered glass coverslips in 6-well culture dishes in the serum-containing medium. Half of the cells was treated with 2 μM CP2 at the moment of plating and kept under that treatment till the day of analysis. Cells were cultured for 3 days, then medium was switched to Neurobasal (NB, cholesterol-free) medium, and cells were cultured for additional 9 days in the presence of CP2. Every third day after switch 1 ml of medium in the cell culture dish was replaced with 1 ml of fresh NB medium (CP2 was kept at the 2 μM concentration all the time). Cells were fixed with 4% paraformaldehyde (PFA) on day 3, 6, and 13 after plating. Coverslips were washed 3 times with PBS, incubated 30 min with glycine (75 mg in 100 ml of PBS), and filipin solution (100 μg/ml, Polysciences, Inc., Warrington, PA) was applied for 30 min at room temperature. Cells were washed in PBS and immediately observed under the microscope (LSM 510, C. Zeiss) using 100× magnification.

Untreated neurons and neurons treated with 2 μM CP2 were compared side by side for intensity of filipin staining. Five to ten cells were imaged for every point. Experiments with other TP compounds using different concentrations were performed the same way.

### Internalization of fluorescent transferrin (Tfn) and lactosylceramide (LacCer)

C_5_-BODIPY- fatty acid labeled analog of lactosylceramide (LacCer) and Alexa Fluor 594 -labeled transferring (Tfn) were from Molecular Probes. Striatal neurons 6 days after plating on poly-L-ornithine coated glass coverslips were washed with HMEM (10 mM HEPES-buffered MEM) at room temperature, and then incubated with BODIPY-LacCer for 10 min at 37°C in the incubator with 5% CO_2 _to induce endocytosis. After incubation, the medium was replaced with ice-cold HMEM without glucose, and the culture dishes were transferred to a 10°C bath. Fluorescent lipid present at the cell surface was removed by incubating the cells (six times, 10 min each) with 5% fatty acid free BSA in HMEM without glucose at 10°C. For other experiments, cells were incubated with 7.5 μg/ml Alexa Fluor 594-labeled Tfn for 10 min at 37°C. Excess of fluorescent marker at the cell surface was removed by acid stripping (HMEM, pH 3). Cells were imaged using confocal laser scanning microscope LSM 510 (Carl Zeiss) with 100× or 63× oil DIC objective (1.4 na) with optical section set to ~0.5 μm.

Control and HD72 non-treated and 2- and 5-μm CP2 treated cells were examined. Neurons were kept under CP2 from the first day of plating till the internalization experiments. Cells were imaged side-by-side using the same settings. Image analysis was done using LSM 510 Physiology software. Student t-test was applied to determine statistical significance between fluorescence readings. Ten to fifteen neurons from three independent platings were taken into analysis.

## Abbreviations

(HD): Huntington's disease; (htt): huntingtin; (mhtt): mutant huntingtin; (TP): tricyclic pyrone; (APP): amyloid β protein precursor; (AD): Alzheimer's disease; (LacCer): lactosylceramide; (IBs): inclusion bodies; (NINDS): National Institute of Neurological Disorders and Stroke; (CREB): cAMP-response element binding protein; (HAP1): huntingtin-associated protein-1; (HIP1): huntingtin interacting protein-1; (Sp1): specificity protein 1; (GFP): green fluorescent protein; (DIC): days in culture; (Tfn): transferrin; (ACAT): acyl-CoA:cholesterol acyltransferase; (NB): neurobasal; (cav-1): caveolin-1; (HMPA): hexamethylphosphoramide; (LDA): lithium diisopropylamide; (THF): tetrahydrofuran; (TFA): trifluoroacetic acid; (Ac_2_O): acetic anhydride; (MsCl): methanesulfonyl chloride.

## Authors' contributions

ET designed and carried out all experiments in primary neuronal cultures, performed imaging and analysis, and drafted the manuscript. SR carried out all experiments in the synthesis of CP2 and TP compounds. CTM -intellectually contributed to the design and analysis of data, critically revised manuscript for final submission. DHH designed CP2 and TP compounds, directed the research project, and drafted the manuscript with ET.
